# Cases of Fractured Sternum, with Observations

**Published:** 1807-09

**Authors:** M. Sabatier

**Affiliations:** Surgeon to the Hotel des Invalides, at Paris


*Cases of Fractured Sternum, with Observations*; by M. SABATIER, Surgeon to the Hotel des Invalides, at Paris.

WHEN the situation of the Sternum, and the thinness of the teguments which cover it, are considered, it might naturally be supposed, that this bone would be very liable to fractures, more especially as its substance, not being extremely compact, can oppose little resistance to external injuries. This, however, is a case of rare occurrence, in comparison with similar accidents sustained by other bones. Few writers on Surgery, not even Hippocrates himself, have treated of fractures of the sternum; while by others it has only been slightly noticed, and compared with those of the scapula, and ossa iliûm.

I. L. Petit and Duverney afford an exception to this remark. The former of these gentlemen, in particular, to whom we are indebted for many improvements in the practice of surgery, has greatly contributed to elucidate this subject. According to him, fractures of the sternum bear much analogy to those of the cranium, and frequently require similar operations. He observes, that when a portion of the bone is displaced, in such a manner as to produce a dangerous pressure on the organs contained in the chest, as well as in cases apparently less formidable, wherein the osseous parts are not depressed; this accident has been attended with effusions, the consequences of which are equally to be dreaded. Martiniére has adopted these principles, in a Paper inserted in the fourth volume of the Memoirs of the Academy of Surgery, where he shews the propriety of applying the trepan in cases of fractured sternum, whether produced by falls, gun-shot wounds, &c.

This operation is fortunately, however, not always necessary, as the two following cases, which fell under my own observation, fully evince.

A middle-aged man, of a robust and sanguine temperment, was run over by a heavy loaded carriage, the wheel of of which passed over his chest from the right side to the left, in consequence of which the sternum, and two of the true ribs, on the left side, were fractured. He instantly vomited a large quantity of blood, and became so weak, that his life was despaired of; a crepitation was heard within the chest, which evidently proceeded from the friction of the fractured portions of the sternum against each other. The inferior portion was pushed forward at each inspiration, and carried backwards during every expiration, while the superior portion remained relatively immovable. I vainly endeavoured to retain them in a proper position by means of pressure, with the hands, to the inferior and anterior parts of the chest. The agony of the patient was, however, augmented, and the difficulty of respiration became so great, that it could not be persevered in above a few minutes. I was therefore obliged to content myself with applying a loose bandage to confine the compresses, which were dipped in a mixture of *oleum hyperici* and ardent spirits, and placed over the whole extent of the contusion. As the pulse was extremely feeble, and the extremities cold, I ordered the patient to be covered up with warm blankets, which very soon recalled the natural heat of the body, and permitted me to employ the lancet. The bleeding was repeated seven times in the course of three days; at the end of which he breathed with so much greater freedom, that he could continue in bed. It may be proper, however, to mention that he remained in some measure in a sitting posture, and so much bent forward, that it was necessary to support him by means of a chair placed flat on the bed and covered with pillows. This was indeed the only position which he could endure for a moment, during the first eight days, through the whole course of which he was restricted to water gruel and mild mucilaginous drinks. By this treatment, the dyspnœa and the pains in the chest became gradually relieved; he occasionally obtained some short intervals of repose; the expectoration was less mixed with blood; the crepitation diminished, and in a short time could no longer be heard. At the termination of three weeks from the accident, the situation of the patient became much improved, but his health was not fully re-established till two or three months afterwards. He continued, however, to experience a slight difficulty of breathing, though on inspection it was scarcely possible to discover any vestiges of the fracture.

From the extensive nature of the injury, I experienced great uneasiness respecting the event. At first I dreaded lest the lungs, the pericardium, and the heart had been bruised, or lacerated, as happened in two or three of the cases related by M. Duverney, and that the life of my patient would terminate in a few hours; and even after such immediate danger was no longer to be apprehended, I conceived that an accumulation of blood or pus might take place opposite the sternum, or in the cells of the mediastinum, and that it would be necessary to lay bare a portion of the bone, to afford a free exit to the effused matter, in case it did not escape through the interstices of the fractured parts, or to have recourse to the employment of the trepan, if these openings were not sufficient. A short time, however, happily proved this conjecture was also unfounded; but the motion of the inferior portion of the bone, and the crepitation, which continued sufficiently audible, rendered me uneasy, lest the fractured bones might not unite, and that a greater difficulty of breathing would remain than that which the patient had yet experienced. It is well known, that in order to facilitate the union of fractured bones, absolute rest is necessary; and, that if this condition be not fulfilled, the cure is not only greatly retarded, but sometimes wholly prevented. Nevertheless, Nature in the present instance surmounted circumstances the most unfavourable; and if a complete callus was not produced, as is most frequently the case, at least the osseous parts became consolidated in such a manner, that no farther motion was perceivable, and the functions of the thoracic viscera were performed as usual.

Since witnessing the above case, which occurred some years ago, I have had occasion to observe the efforts of Nature in an instance of fractured ribs, which, though very different from the one just related, affords an equal proof of her power in circumstances which preclude the interference of art.

A young robust coachman having fallen from his seat, one of the wheels run over his chest from left to right, by which accident the last two true ribs, on the left side, were fractured; the undermost of them in two places, so that the intermediate portion of bone was completely detached. Its motion was very considerable, and it was evidently carried towards the inside of the chest in the act of inspiration, and again thrown outwards during expiration. The consequences produced by this singular fracture were not, however, very formidable, and the patient was completely cured in the space of a short time.

A second case of fractured sternum, which lately occurred in my practice, did not prove equally fortunate in its its termination as the first. A man upwards of 60 years of age, after having been violently bruised and beaten with the fist, was thrown by the individuals who assaulted him into a ditch thirty feet in depth. He fell on his back, and the shock was so great, as wholly to deprive him of the power of motion, and he remained in the same position from eight o'clock in the evening until the same hour on the following morning. He was removed with much difficulty from Vincennes, where the accident happened, to the Hôtel des Invalides. On being brought there, he was extremely weak; his respiration was convulsive; he expectorated a considerable quantity of blood, and complained of pain and soreness over all his body, but particularly in the region of the sternum. One of my pupils, who first saw him, perceived on examination that the sternum was fractured transversely, at the place where the first bone forms a junction with the second, and that this had been forced beneath the other. He endeavoured by the employment of lateral pressure to restore the bones to their natural situation; but not succeeding in his attempts, he bled the patient, and applied over the chest, and particularly to the lower part of the sternum, thick compresses, previously soaked in an embrocation, and supported by means of a roller passed round the body, and drawn somewhat tight at the lower part. Things were in this state, when I arrived. His respiration was performed in so singular a manner, and his pulse so extremely languid and feeble, that I conceived it impossible to afford him any assistance. Contrary to my expectations, however, I found him still living on the following morning; his pulse had become somewhat stronger, his respiration was not quite so laborious, and he had expectorated a considerable quantity of mucous matter. He still, however, appeared in very imminent danger. Besides a renewal of the embrocation, I prescribed for him a balsamic and pectoral decoction. For a few days, the symptoms continued nearly stationary, except that he appeared to suffer less, and to expectorate more easily, so that I began to entertain hopes of his recovery, and that he would acquire a sufficient degree of strength to allow us to put in practice some of the means recommended for raising up the depressed portion of the sternum, when he died on the eighth day from the accident.

On dissection, I found the sternum fractured at the place already mentioned, and the inferior part of the bone not only depressed, but forced in a little way beneath the superior portion. There was a considerable extravasation of blood beneath the integuments, as well as in the substance of the right lobe of the lungs, which every where adhered to the pleura; but this adhesion, it was obvious, had long existed. On the left side, neither the lungs, pericardium, nor heart, had suffered any injury, and there appeared no effusion of blood, which could be attributed to the rupture of the mammary arteries. This rupture is, however, one of the sequelæ most to be dreaded in fractures of the sternum, though even the organs contained in the chest should have suffered no material injury. The most remarkable circumstance in the present case, was the relative situation of the fractured bones. So far as I am acquainted, no writer on this subject has noticed it; because the bones of the sternum seldom overlap each other, even when the fracture is complete and accompanied with depression. The action of the intercostal muscles, which tends to diminish he interval between the ribs, and bring them together, should seem, however, capable of frequently producing this effect. It is easy to conceive that in such a case, the crepitation, which is óne of the most certain signs of fractured sternum, and which, in my first patient, was distinctly audible on the least motion of the chest, without any pressure being employed to the injured parts, could not occur; and that if the external teguments were so much tumefied, that we had no other method of determining the nature of the injury but by the touch, we might readily fall into error, or form a false judgment respecting the real state of the part. I am also of opinion, that the processes generally recommended to restore the depressed bone to its natural situation are wholly unavailing. For how in fact can lateral pressure on the chest succeed in replacing it, though even the spine be bent backwards, with the intention of forcibly separating the superior from the inferior portion of the sternum? Neither is the method more available which some recommend, of exposing the fractured part, and of raising the depressed bone by means of an elevator, &c.

In cases wherein the overlapping is not so great as in the present instance, it might perhaps be possible, by this or similar means, to bring the divided portions of the bones into contact, or juxta position, with each other, but it would be wholly impracticable to retain them in that situation; and it is much to be feared, that the cause originally productive of the derangement, which we have pointed out, might displace them anew, especially if the fracture was in a very oblique direction.

The conclusion which results from these observations, is, it must be confessed, extremely depressing; since it is evident, that certain fractures of the sternum are beyond the power of art to remedy, and that they even sometimes prove mortal in cases wherein the internal viscera have sustained no very material injury. Instances of this last kind, must, however, have occurred more frequently, previous to the period at which Martiniere published his reflections on the application of the trepan to the sternum; as in fact, internal effusions, which require this operation, often occur without any displacement of the bones, and when they have merely sustained contusion.

It may be here proper to remark, that the case above related, exhibits a singular and incontestible instance of repercussion in the chest. This frequently happens in injuries of the head, and the cause is too well known and too intimately connected with the changes that spherical bodies undergo when they are struck, to render it necessary to enter into any reasoning on its possibility. It is well known that instances of a similar kind occur in other parts of the body; and that the injuries occasioned by strokes or falls frequently appear at a great distance from the parts on which the blow had been inflicted. In this last case a continuity of motion, or concussion, is communicated to the soft parts contained in the large cavities of the body. But in the present it is very different, and is produced in the same manner as fractures of the cranium frequently occur in the side opposite to that which received the stroke, and which are not an effect of continuity of motion, but proceed from that change of form which is the necessary result of repercussion on spherical bodies, but which is only evident when this re percussion is extremely violent, and attended with consequences equally marked as those of the case under consideration.

If any doubts remain on this subject, they may be easily resolved by comparing my observations with those in the fourth volume of the *Prix de l'Académie de Chirurgie*, in a memoir by Basile, respecting re-percussion in fractures in other parts of the body than the head, and in which he examines whether a fracture can occur in the sternum in consequence of a fall on the back. In the instance to which he alludes, the patient had fallen from a height of fifty feet, and there was no external mark indicating that he had pitched on the forepart of the chest. On the contrary, it was affirmed, that his back had struck a projecting portion of a scaffold before he reached the ground.

From

From this circumstance, the author takes occasion to suggest a curious explanation of the fact; he conceives the sternum must have been ruptured by the simultaneous action of the muscles which are inserted into the superior and inferior portion of this bone, and which had contracted with such force as to prevent the body bending backwards at the moment of receiving the shock. Be this however as it may, a species of re-percussion must still have been produced, though differing as to the cause from that which it appeared to have in the case I have narrated.

On the whole it must therefore appear equally necessary to extend our examination to parts at a distance from those which have been struck, in cases of blows or falls on the chest, as in those of the cranium, since similar injuries, and probably from the same cause, may occur in both instances.

